# Supplementary material for: Smooth Interpolating Curves with Local Control and Monotone Alternating Curvature
Source: Comput Graph Forum. 2022 Oct 6;41(5):25–38. doi: 10.1111/cgf.14600 (PMC9827861; doi:10.1111/cgf.14600)
Supplement: Supplementary file 1 — Supplement Material [file CGF-41-25-s001.zip › Local-Smooth-Interpolating-MonoCurvature/extern/clothoids/docs/api-cpp/program_listing_file_G2lib_intersect.cc.html]

Program Listing for File G2lib\_intersect.cc — Clothoids v2.0.9

### Navigation

- index
- toc
- Clothoids »
- Program Listing for File G2lib\_intersect.cc

# Program Listing for File G2lib\_intersect.cc¶

↰ Return to documentation for file (`G2lib_intersect.cc`)

```
/*--------------------------------------------------------------------------*\
 |                                                                          |
 |  Copyright (C) 2017                                                      |
 |                                                                          |
 |         , __                 , __                                        |
 |        /|/  \               /|/  \                                       |
 |         | __/ _   ,_         | __/ _   ,_                                |
 |         |   \|/  /  |  |   | |   \|/  /  |  |   |                        |
 |         |(__/|__/   |_/ \_/|/|(__/|__/   |_/ \_/|/                       |
 |                           /|                   /|                        |
 |                           \|                   \|                        |
 |                                                                          |
 |      Enrico Bertolazzi                                                   |
 |      Dipartimento di Ingegneria Industriale                              |
 |      Universita` degli Studi di Trento                                   |
 |      email: enrico.bertolazzi@unitn.it                                   |
 |                                                                          |
\*--------------------------------------------------------------------------*/

#include "Clothoids.hh"

#ifdef __clang__
#pragma clang diagnostic ignored "-Wglobal-constructors"
#pragma clang diagnostic ignored "-Wexit-time-destructors"
#endif

namespace G2lib {

  using std::map;
  using std::pair;

  using std::numeric_limits;
  using std::fpclassify;
  using std::lower_bound;
  using std::abs;
  using std::sqrt;

  /*\
   |   _       _                          _
   |  (_)_ __ | |_ ___ _ __ ___  ___  ___| |_
   |  | | '_ \| __/ _ \ '__/ __|/ _ \/ __| __|
   |  | | | | | ||  __/ |  \__ \  __/ (__| |_
   |  |_|_| |_|\__\___|_|  |___/\___|\___|\__|
  \*/

  #ifndef DOXYGEN_SHOULD_SKIP_THIS

  typedef pair<CurveType,CurveType> Ppair;

  // check if compiler is C++11
  #ifdef G2LIB_USE_CXX11
  static map<Ppair,CurveType> const promote_map = {
    {Ppair( G2LIB_LINE, G2LIB_LINE ),          G2LIB_LINE},
    {Ppair( G2LIB_LINE, G2LIB_CIRCLE ),        G2LIB_CIRCLE},
    {Ppair( G2LIB_LINE, G2LIB_CLOTHOID ),      G2LIB_CLOTHOID},
    {Ppair( G2LIB_LINE, G2LIB_BIARC ),         G2LIB_BIARC_LIST},
    {Ppair( G2LIB_LINE, G2LIB_BIARC_LIST ),    G2LIB_BIARC_LIST},
    {Ppair( G2LIB_LINE, G2LIB_CLOTHOID_LIST ), G2LIB_CLOTHOID_LIST},
    {Ppair( G2LIB_LINE, G2LIB_POLYLINE ),      G2LIB_POLYLINE},

    {Ppair( G2LIB_CIRCLE, G2LIB_LINE ),          G2LIB_CIRCLE},
    {Ppair( G2LIB_CIRCLE, G2LIB_CIRCLE ),        G2LIB_CIRCLE},
    {Ppair( G2LIB_CIRCLE, G2LIB_CLOTHOID ),      G2LIB_CLOTHOID},
    {Ppair( G2LIB_CIRCLE, G2LIB_BIARC ),         G2LIB_BIARC_LIST},
    {Ppair( G2LIB_CIRCLE, G2LIB_BIARC_LIST ),    G2LIB_BIARC_LIST},
    {Ppair( G2LIB_CIRCLE, G2LIB_CLOTHOID_LIST ), G2LIB_CLOTHOID_LIST},
    {Ppair( G2LIB_CIRCLE, G2LIB_POLYLINE ),      G2LIB_CLOTHOID_LIST},

    {Ppair( G2LIB_BIARC, G2LIB_LINE ),          G2LIB_CLOTHOID_LIST},
    {Ppair( G2LIB_BIARC, G2LIB_CIRCLE ),        G2LIB_CLOTHOID_LIST},
    {Ppair( G2LIB_BIARC, G2LIB_CLOTHOID ),      G2LIB_CLOTHOID_LIST},
    {Ppair( G2LIB_BIARC, G2LIB_BIARC ),         G2LIB_BIARC},
    {Ppair( G2LIB_BIARC, G2LIB_BIARC_LIST ),    G2LIB_BIARC_LIST},
    {Ppair( G2LIB_BIARC, G2LIB_CLOTHOID_LIST ), G2LIB_CLOTHOID_LIST},
    {Ppair( G2LIB_BIARC, G2LIB_POLYLINE ),      G2LIB_CLOTHOID_LIST},

    {Ppair( G2LIB_CLOTHOID, G2LIB_LINE ),          G2LIB_CLOTHOID},
    {Ppair( G2LIB_CLOTHOID, G2LIB_CIRCLE ),        G2LIB_CLOTHOID},
    {Ppair( G2LIB_CLOTHOID, G2LIB_CLOTHOID ),      G2LIB_CLOTHOID},
    {Ppair( G2LIB_CLOTHOID, G2LIB_BIARC ),         G2LIB_CLOTHOID_LIST},
    {Ppair( G2LIB_CLOTHOID, G2LIB_BIARC_LIST ),    G2LIB_CLOTHOID_LIST},
    {Ppair( G2LIB_CLOTHOID, G2LIB_CLOTHOID_LIST ), G2LIB_CLOTHOID_LIST},
    {Ppair( G2LIB_CLOTHOID, G2LIB_POLYLINE ),      G2LIB_CLOTHOID_LIST},

    {Ppair( G2LIB_CLOTHOID_LIST, G2LIB_LINE ),          G2LIB_CLOTHOID_LIST},
    {Ppair( G2LIB_CLOTHOID_LIST, G2LIB_CIRCLE ),        G2LIB_CLOTHOID_LIST},
    {Ppair( G2LIB_CLOTHOID_LIST, G2LIB_CLOTHOID ),      G2LIB_CLOTHOID_LIST},
    {Ppair( G2LIB_CLOTHOID_LIST, G2LIB_BIARC ),         G2LIB_CLOTHOID_LIST},
    {Ppair( G2LIB_CLOTHOID_LIST, G2LIB_BIARC_LIST ),    G2LIB_CLOTHOID_LIST},
    {Ppair( G2LIB_CLOTHOID_LIST, G2LIB_CLOTHOID_LIST ), G2LIB_CLOTHOID_LIST},
    {Ppair( G2LIB_CLOTHOID_LIST, G2LIB_POLYLINE ),      G2LIB_CLOTHOID_LIST},

    {Ppair( G2LIB_POLYLINE, G2LIB_LINE ),          G2LIB_POLYLINE},
    {Ppair( G2LIB_POLYLINE, G2LIB_CIRCLE ),        G2LIB_CLOTHOID_LIST},
    {Ppair( G2LIB_POLYLINE, G2LIB_CLOTHOID ),      G2LIB_CLOTHOID_LIST},
    {Ppair( G2LIB_POLYLINE, G2LIB_BIARC ),         G2LIB_CLOTHOID_LIST},
    {Ppair( G2LIB_POLYLINE, G2LIB_BIARC_LIST ),    G2LIB_CLOTHOID_LIST},
    {Ppair( G2LIB_POLYLINE, G2LIB_CLOTHOID_LIST ), G2LIB_CLOTHOID_LIST},
    {Ppair( G2LIB_POLYLINE, G2LIB_POLYLINE ),      G2LIB_POLYLINE}
  };
  #else
    static map<Ppair,CurveType> promote_map;
    static
    void
    init_promote_map() {
      static bool done = false;
      if ( done ) return;
      promote_map[ Ppair( G2LIB_LINE, G2LIB_LINE ) ]          = G2LIB_LINE;
      promote_map[ Ppair( G2LIB_LINE, G2LIB_CIRCLE ) ]        = G2LIB_CIRCLE;
      promote_map[ Ppair( G2LIB_LINE, G2LIB_CLOTHOID ) ]      = G2LIB_CLOTHOID;
      promote_map[ Ppair( G2LIB_LINE, G2LIB_BIARC ) ]         = G2LIB_BIARC_LIST;
      promote_map[ Ppair( G2LIB_LINE, G2LIB_BIARC_LIST ) ]    = G2LIB_BIARC_LIST;
      promote_map[ Ppair( G2LIB_LINE, G2LIB_CLOTHOID_LIST ) ] = G2LIB_CLOTHOID_LIST;
      promote_map[ Ppair( G2LIB_LINE, G2LIB_POLYLINE ) ]      = G2LIB_POLYLINE;

      promote_map[ Ppair( G2LIB_CIRCLE, G2LIB_LINE ) ]          = G2LIB_CIRCLE;
      promote_map[ Ppair( G2LIB_CIRCLE, G2LIB_CIRCLE ) ]        = G2LIB_CIRCLE;
      promote_map[ Ppair( G2LIB_CIRCLE, G2LIB_CLOTHOID ) ]      = G2LIB_CLOTHOID;
      promote_map[ Ppair( G2LIB_CIRCLE, G2LIB_BIARC ) ]         = G2LIB_BIARC_LIST;
      promote_map[ Ppair( G2LIB_CIRCLE, G2LIB_BIARC_LIST ) ]    = G2LIB_BIARC_LIST;
      promote_map[ Ppair( G2LIB_CIRCLE, G2LIB_CLOTHOID_LIST ) ] = G2LIB_CLOTHOID_LIST;
      promote_map[ Ppair( G2LIB_CIRCLE, G2LIB_POLYLINE ) ]      = G2LIB_CLOTHOID_LIST;

      promote_map[ Ppair( G2LIB_BIARC, G2LIB_LINE ) ]          = G2LIB_CLOTHOID_LIST;
      promote_map[ Ppair( G2LIB_BIARC, G2LIB_CIRCLE ) ]        = G2LIB_CLOTHOID_LIST;
      promote_map[ Ppair( G2LIB_BIARC, G2LIB_CLOTHOID ) ]      = G2LIB_CLOTHOID_LIST;
      promote_map[ Ppair( G2LIB_BIARC, G2LIB_BIARC ) ]         = G2LIB_BIARC;
      promote_map[ Ppair( G2LIB_BIARC, G2LIB_BIARC_LIST ) ]    = G2LIB_BIARC_LIST;
      promote_map[ Ppair( G2LIB_BIARC, G2LIB_CLOTHOID_LIST ) ] = G2LIB_CLOTHOID_LIST;
      promote_map[ Ppair( G2LIB_BIARC, G2LIB_POLYLINE ) ]      = G2LIB_CLOTHOID_LIST;

      promote_map[ Ppair( G2LIB_CLOTHOID, G2LIB_LINE ) ]          = G2LIB_CLOTHOID;
      promote_map[ Ppair( G2LIB_CLOTHOID, G2LIB_CIRCLE ) ]        = G2LIB_CLOTHOID;
      promote_map[ Ppair( G2LIB_CLOTHOID, G2LIB_CLOTHOID ) ]      = G2LIB_CLOTHOID;
      promote_map[ Ppair( G2LIB_CLOTHOID, G2LIB_BIARC ) ]         = G2LIB_CLOTHOID_LIST;
      promote_map[ Ppair( G2LIB_CLOTHOID, G2LIB_BIARC_LIST ) ]    = G2LIB_CLOTHOID_LIST;
      promote_map[ Ppair( G2LIB_CLOTHOID, G2LIB_CLOTHOID_LIST ) ] = G2LIB_CLOTHOID_LIST;
      promote_map[ Ppair( G2LIB_CLOTHOID, G2LIB_POLYLINE ) ]      = G2LIB_CLOTHOID_LIST;

      promote_map[ Ppair( G2LIB_CLOTHOID_LIST, G2LIB_LINE ) ]          = G2LIB_CLOTHOID_LIST;
      promote_map[ Ppair( G2LIB_CLOTHOID_LIST, G2LIB_CIRCLE ) ]        = G2LIB_CLOTHOID_LIST;
      promote_map[ Ppair( G2LIB_CLOTHOID_LIST, G2LIB_CLOTHOID ) ]      = G2LIB_CLOTHOID_LIST;
      promote_map[ Ppair( G2LIB_CLOTHOID_LIST, G2LIB_BIARC ) ]         = G2LIB_CLOTHOID_LIST;
      promote_map[ Ppair( G2LIB_CLOTHOID_LIST, G2LIB_BIARC_LIST ) ]    = G2LIB_CLOTHOID_LIST;
      promote_map[ Ppair( G2LIB_CLOTHOID_LIST, G2LIB_CLOTHOID_LIST ) ] = G2LIB_CLOTHOID_LIST;
      promote_map[ Ppair( G2LIB_CLOTHOID_LIST, G2LIB_POLYLINE ) ]      = G2LIB_CLOTHOID_LIST;

      promote_map[ Ppair( G2LIB_POLYLINE, G2LIB_LINE ) ]          = G2LIB_POLYLINE;
      promote_map[ Ppair( G2LIB_POLYLINE, G2LIB_CIRCLE ) ]        = G2LIB_CLOTHOID_LIST;
      promote_map[ Ppair( G2LIB_POLYLINE, G2LIB_CLOTHOID ) ]      = G2LIB_CLOTHOID_LIST;
      promote_map[ Ppair( G2LIB_POLYLINE, G2LIB_BIARC ) ]         = G2LIB_CLOTHOID_LIST;
      promote_map[ Ppair( G2LIB_POLYLINE, G2LIB_BIARC_LIST ) ]    = G2LIB_CLOTHOID_LIST;
      promote_map[ Ppair( G2LIB_POLYLINE, G2LIB_CLOTHOID_LIST ) ] = G2LIB_CLOTHOID_LIST;
      promote_map[ Ppair( G2LIB_POLYLINE, G2LIB_POLYLINE ) ]      = G2LIB_POLYLINE;
      done = true;
    }
  #endif

  #endif

  // - - - - - - - - - - - - - - - - - - - - - - - - - - - - - - - - - - - - - -

  bool
  collision( BaseCurve const & obj1, BaseCurve const & obj2 ) {
    G2LIB_DEBUG_MESSAGE(
      "collision {} with {} using {}\n",
      CurveType_name[obj1.type()],
      CurveType_name[obj2.type()],
      CurveType_name[promote_map.at(Ppair(obj1.type(),obj2.type()))]
    );

    bool ok = false;
    switch ( promote_map.at(Ppair(obj1.type(),obj2.type())) ) {
    case G2LIB_LINE:
      {
        LineSegment L1( obj1 );
        LineSegment L2( obj2 );
        ok = L1.collision( L2 );
      }
      break;
    case G2LIB_CIRCLE:
      {
        CircleArc C1( obj1 );
        CircleArc C2( obj2 );
        ok = C1.collision( C2 );
      }
      break;
    case G2LIB_CLOTHOID:
      {
        ClothoidCurve C1( obj1 );
        ClothoidCurve C2( obj2 );
        ok = C1.collision( C2 );
      }
      break;
    case G2LIB_BIARC:
      {
        Biarc B1( obj1 );
        Biarc B2( obj2 );
        ok = B1.collision( B2 );
      }
      break;
    case G2LIB_BIARC_LIST:
      {
        BiarcList BL1( obj1 );
        BiarcList BL2( obj2 );
        ok = BL1.collision( BL2 );
      }
      break;
    case G2LIB_CLOTHOID_LIST:
      {
        ClothoidList CL1( obj1 );
        ClothoidList CL2( obj2 );
        ok = CL1.collision( CL2 );
      }
      break;
    case G2LIB_POLYLINE:
      {
        PolyLine PL1( obj1 );
        PolyLine PL2( obj2 );
        ok = PL1.collision( PL2 );
      }
      break;
    }
    return ok;
  }

  // - - - - - - - - - - - - - - - - - - - - - - - - - - - - - - - - - - - - - -

  bool
  collision_ISO(
    BaseCurve const & obj1,
    real_type         offs1,
    BaseCurve const & obj2,
    real_type         offs2
  ) {
    #ifndef G2LIB_USE_CXX11
    init_promote_map();
    #endif

    G2LIB_DEBUG_MESSAGE(
      "collision (offs) {} with {} using {}\n",
      CurveType_name[obj1.type()],
      CurveType_name[obj2.type()],
      CurveType_name[promote_map.at(Ppair(obj1.type(),obj2.type()))]
    );

    bool ok = false;
    switch ( promote_map.at(Ppair(obj1.type(),obj2.type())) ) {
    case G2LIB_LINE:
      {
        LineSegment L1( obj1 );
        LineSegment L2( obj2 );
        ok = L1.collision_ISO( offs1, L2, offs2 );
      }
      break;
    case G2LIB_CIRCLE:
      {
        CircleArc C1( obj1 );
        CircleArc C2( obj2 );
        ok = C1.collision_ISO( offs1, C2, offs2 );
      }
      break;
    case G2LIB_CLOTHOID:
      {
        ClothoidCurve C1( obj1 );
        ClothoidCurve C2( obj2 );
        ok = C1.collision_ISO( offs1, C2, offs2 );
      }
      break;
    case G2LIB_BIARC:
      {
        Biarc B1( obj1 );
        Biarc B2( obj2 );
        ok = B1.collision_ISO( offs1, B2, offs2 );
      }
      break;
    case G2LIB_BIARC_LIST:
      {
        BiarcList BL1( obj1 );
        BiarcList BL2( obj2 );
        ok = BL1.collision_ISO( offs1, BL2, offs2 );
      }
      break;
    case G2LIB_CLOTHOID_LIST:
      {
        ClothoidList CL1( obj1 );
        ClothoidList CL2( obj2 );
        ok = CL1.collision_ISO( offs1, CL2, offs2 );
      }
      break;
    case G2LIB_POLYLINE:
      {
        PolyLine PL1( obj1 );
        PolyLine PL2( obj2 );
        ok = PL1.collision_ISO( offs1, PL2, offs2 );
      }
    }
    return ok;
  }

  // - - - - - - - - - - - - - - - - - - - - - - - - - - - - - - - - - - - - - -

  void
  intersect(
    BaseCurve const & obj1,
    BaseCurve const & obj2,
    IntersectList   & ilist,
    bool              swap_s_vals
  ) {
    #ifndef G2LIB_USE_CXX11
    init_promote_map();
    #endif

    G2LIB_DEBUG_MESSAGE(
      "intersect {} with {} using {}\n",
      CurveType_name[obj1.type()],
      CurveType_name[obj2.type()],
      CurveType_name[promote_map.at(Ppair(obj1.type(),obj2.type()))]
    );

    switch ( promote_map.at(Ppair(obj1.type(),obj2.type())) ) {
    case G2LIB_LINE:
      {
        LineSegment L1( obj1 );
        LineSegment L2( obj2 );
        L1.intersect( L2, ilist, swap_s_vals );
      }
      break;
    case G2LIB_CIRCLE:
      {
        CircleArc C1( obj1 );
        CircleArc C2( obj2 );
        C1.intersect( C2, ilist, swap_s_vals );
      }
      break;
    case G2LIB_CLOTHOID:
      {
        ClothoidCurve C1( obj1 );
        ClothoidCurve C2( obj2 );
        C1.intersect( C2, ilist, swap_s_vals );
      }
      break;
    case G2LIB_BIARC:
      {
        Biarc B1( obj1 );
        Biarc B2( obj2 );
        B1.intersect( B2, ilist, swap_s_vals );
      }
      break;
    case G2LIB_BIARC_LIST:
      {
        BiarcList BL1( obj1 );
        BiarcList BL2( obj2 );
        BL1.intersect( BL2, ilist, swap_s_vals );
      }
      break;
    case G2LIB_CLOTHOID_LIST:
      {
        ClothoidList CL1( obj1 );
        ClothoidList CL2( obj2 );
        CL1.intersect( CL2, ilist, swap_s_vals );
      }
      break;
    case G2LIB_POLYLINE:
      {
        PolyLine PL1( obj1 );
        PolyLine PL2( obj2 );
        PL1.intersect( PL2, ilist, swap_s_vals );
      }
      break;
    }
  }

  // - - - - - - - - - - - - - - - - - - - - - - - - - - - - - - - - - - - - - -

  void
  intersect_ISO(
    BaseCurve const & obj1,
    real_type         offs1,
    BaseCurve const & obj2,
    real_type         offs2,
    IntersectList   & ilist,
    bool              swap_s_vals
  ) {
    #ifndef G2LIB_USE_CXX11
    init_promote_map();
    #endif

    G2LIB_DEBUG_MESSAGE(
      "intersect (offs) {} with {} using {}\n",
      CurveType_name[obj1.type()],
      CurveType_name[obj2.type()],
      CurveType_name[promote_map.at(Ppair(obj1.type(),obj2.type()))]
    );

    switch ( promote_map.at(Ppair(obj1.type(),obj2.type())) ) {
    case G2LIB_LINE:
      {
        LineSegment L1( obj1 );
        LineSegment L2( obj2 );
        L1.intersect_ISO( offs1, L2, offs2, ilist, swap_s_vals );
      }
      break;
    case G2LIB_CIRCLE:
      {
        CircleArc C1( obj1 );
        CircleArc C2( obj2 );
        C1.intersect_ISO( offs1, C2, offs2, ilist, swap_s_vals );
      }
      break;
    case G2LIB_CLOTHOID:
      {
        ClothoidCurve C1( obj1 );
        ClothoidCurve C2( obj2 );
        C1.intersect_ISO( offs1, C2, offs2, ilist, swap_s_vals );
      }
      break;
    case G2LIB_BIARC:
      {
        Biarc B1( obj1 );
        Biarc B2( obj2 );
        B1.intersect_ISO( offs1, B2, offs2, ilist, swap_s_vals );
      }
      break;
    case G2LIB_BIARC_LIST:
      {
        BiarcList BL1( obj1 );
        BiarcList BL2( obj2 );
        BL1.intersect_ISO( offs1, BL2, offs2, ilist, swap_s_vals );
      }
      break;
    case G2LIB_CLOTHOID_LIST:
      {
        ClothoidList CL1( obj1 );
        ClothoidList CL2( obj2 );
        CL1.intersect_ISO( offs1, CL2, offs2, ilist, swap_s_vals );
      }
      break;
    case G2LIB_POLYLINE:
      {
        PolyLine PL1( obj1 );
        PolyLine PL2( obj2 );
        PL1.intersect_ISO( offs1, PL2, offs2, ilist, swap_s_vals );
      }
      break;
    }
  }
}

// EOF: G2lib_intersect.cc
```

### Quick search

### Table of Contents

- Matlab Interface Manual
- C++ API
- MATLAB API

«
hide menu

menu
sidebar
»

### Navigation

- index
- toc
- Clothoids »
- Program Listing for File G2lib\_intersect.cc

© Copyright 2021, Enrico Bertolazzi and Marco Frego.
Created using Sphinx 4.2.0.
